# Simplified strategy for rapid first-line screening of fragile X syndrome: closed-tube triplet-primed PCR and amplicon melt peak analysis

**DOI:** 10.1017/erm.2015.5

**Published:** 2015-05-04

**Authors:** Indhu-Shree Rajan-Babu, Hai-Yang Law, Chui-Sheun Yoon, Caroline G. Lee, Samuel S. Chong

**Affiliations:** 1 Department of Pediatrics, Yong Loo Lin School of Medicine, National University of Singapore, Singapore, Singapore; 2 Department of Pediatric Medicine, KK Women's and Children's Hospital, Singapore, Singapore; 3 Pediatrics Academic Clinical Program, Duke-NUS Graduate Medical School, Singapore; 4 Department of Biochemistry, Yong Loo Lin School of Medicine, National University of Singapore, Singapore, Singapore; 5 Division of Medical Sciences, National Cancer Center, Singapore, Singapore; 6 Cancer and Stem Cell Biology Research Program, Duke-NUS Graduate Medical School, Singapore; 7 Khoo Teck Puat – National University Children's Medical Institute, National University Health System, Singapore, Singapore; 8 Department of Laboratory Medicine, National University Hospital, Singapore, Singapore

## Abstract

Premutation and full-mutation hyperexpansion of CGG-triplets in the X-linked *Fragile X Mental Retardation 1* (*FMR1*) gene have been implicated in fragile X-associated tremor/ataxia syndrome, fragile X-associated primary ovarian insufficiency, and fragile X syndrome (FXS), respectively. The currently available molecular diagnostic tests are either costly or labour-intensive, which prohibits their application as a first-line *FMR1* test in large-scale population-based screening programs. In this study, we demonstrate the utility of a simplified closed-tube strategy for rapid first-line screening of FXS based on melt peak temperature (*T*_m_) analysis of direct triplet-primed polymerase chain reaction amplicons (dTP-PCR MCA). In addition, we also evaluated the correlation between *T*_m_ and CGG-repeat size based on capillary electrophoresis (CE) of dTP-PCR amplicons. The assays were initially tested on 29 *FMR1* reference DNA samples, followed by a blinded validation on 107 previously characterised patient DNA samples. The dTP-PCR MCA produced distinct melt profiles of higher *T*_m_ for samples carrying an expanded allele. Among the samples tested, we also observed a good correlation between *T*_m_ and CGG-repeat size. In the blinded validation study, dTP-PCR MCA accurately classified all normal and expansion carriers, and the *FMR1* genotypic classification of all samples was completely concordant with the previously determined genotypes as well as the dTP-PCR CE results. This simple and cost-effective MCA-based assay may be useful as a first-line FXS screening tool that could rapidly screen out the large majority of unaffected individuals, thus minimising the number of samples that need to be analysed by Southern blot analysis.

## Introduction

Fragile X syndrome (FXS) is the most common inherited form of intellectual disability (ID) affecting ~1/4000 males and ~1/5000 to 1/8000 females (Refs 1, 2) and is caused by the full-mutation (FM, >200 repeats) hyperexpansion of (CGG)_*n*_ triplet-repeat sequence in the 5′-untranslated region of the X-linked *Fragile X Mental Retardation 1* (*FMR1*) gene (Refs 3, 4, 5, 6). FXS is characterised by ID, seizures and other behavioural issues such as anxiety, hyperactivity and autism spectrum disorder (ASD). Symptoms are usually milder in affected females due to X-inactivation and cellular mosaicism (Ref. 7). Premutation (PM) expansions (55–200 repeats), which have been associated with the late-onset *FMR1*-related conditions fragile X-associated tremor/ataxia syndrome (FXTAS) and fragile X-associated primary ovarian insufficiency (FXPOI), occur more frequently, with three recent studies reporting PM prevalence rates of 1/209 females and 1/430 males (Ref. 8), 1/148 females and 1/290 males (Ref. 9) and 1/151 females and 1/468 males (Ref. 10). Approximately 40% of male and 8–16% of female PM carriers will develop FXTAS (Refs 11, 12), with female PM carriers having an additional 20% risk for FXPOI (Refs 13, 14). In addition, a spectrum of medical co-morbidities such as thyroid dysfunction, fibromyalgia, neuropathy, sleep apnea, seizures and hypertension have been observed among PM carriers, particularly in females with FXTAS (Refs 15, 16, 17, 18, 19, 20).

Developmental delay, attention problems, ASD and anxiety have also been reported in some PM individuals (Refs 21, 22). Furthermore, the meiotically unstable nature of expanded *FMR1* alleles predisposes female PM carriers to an increased risk of having FXS-affected offspring, with almost 100% FM transmission risk when the transmitted allele is >90 CGG repeats (Ref. 23). This marked decline in repeat stability among expansion carriers is strongly correlated with the reduction or absence of AGG interruptions within the CGG repeat tract (Refs 24, 25). Normal (NL, 5–44 repeats) and intermediate (IM, 45–54 repeats) alleles do not expand to FM upon transmission, although some IM alleles can gradually expand to FM after multiple generations (Refs 26, 27). However, the key question of whether IM individuals are at risk for PM-related conditions still remains to be addressed (Ref. 2).

According to the FXS testing guidelines, only individuals with a family history of FXS, ASD, developmental delay, learning disabilities or clinical features suggestive of FXS, FXTAS or FXPOI, qualify for *FMR1* molecular testing (Refs 1, 28). There has been enormous interest in evaluating the possibilities of extending fragile X genetic tests to newborns and women of reproductive age so as to detect expansion carriers who are most likely to benefit from early identification (Refs 1, 21, 29, 30, 31, 32). However, for such large-scale screenings to be practicable, cost-effective and technically simpler *FMR1* screening tools are required. Southern blot (SB), the gold standard FXS testing method is still the preferred diagnostic tool for characterising expanded *FMR1* alleles in FM individuals. However, owing to the laborious and low-throughput nature of SB, several polymerase chain reaction (PCR)-based assays have been developed as alternative procedures primarily to minimise or eliminate the need to perform SB on every sample (Refs 33, 34, 35, 36, 37, 38, 39, 40). Most commonly, these studies have employed the traditional repeat-spanning PCR and/or the triplet-repeat primed PCR (TP-PCR). Although the initial limitation of amplifying large GC-rich PM and FM expansions has been overcome (Refs 39, 40), the reliability and upper limit of FM sizing ability of repeat-spanning PCR is contentious. In contrast, TP-PCR methods can reliably detect all FM alleles regardless of the actual FM repeat size. TP-PCR assays facilitate accurate repeat size determination of up to ~200 CGG repeats by virtue of their assay design that incorporates capillary electrophoresis (CE) for analysing the heterogeneous TP-PCR amplicon fragments (Refs 37, 38). However, for newborn and carrier screening applications that involve processing large numbers of samples, methods requiring CE are not ideal due to the high-cost factor associated with the selected approach of amplicon analysis.

We had previously proposed a cost-effective screening platform that coupled TP-PCR with melting curve analysis (MCA) for rapid identification of *FMR1* expansions in both males and females (Ref. 41). This method classified samples as ‘expanded’ or ‘nonexpanded’ based on the temperature at which their melt profiles’ –d*F*/d*T* value dropped to zero or returned to baseline. A higher return to baseline temperature was used as an indicator to confirm the presence of an expanded allele. Therefore, for unambiguous determination of expansion status in samples carrying *FMR1* alleles in the high-IM to low-PM size range, precise discernment of return to baseline temperature is imperative. Reliance on this analysis mode is prone to producing ambiguous results when samples do not display an obvious drop in –d*F*/d*T*. The assay did not generate defined melt peaks, which prohibited identification of melt peak temperature (*T*_m_) and necessitated dependence on return to baseline temperature.

We now describe an improved direct TP-PCR (dTP-PCR) MCA assay that yields remarkably well-defined melt profiles, thus enabling utilisation of the highly reliable melt peak temperature (*T*_m_) instead of the return to baseline temperature to identify expansion carriers. Furthermore, to show the agreement between dTP-PCR MCA classification and the actual *FMR1* genotypes of tested samples, we simultaneously performed a dTP-PCR CE-based sizing assay that was optimised to detect all *FMR1* allelic classes. In addition, we have also demonstrated the sensitivity of both assays in detecting low-level mosaicism for FM allele using artificial DNA mixtures.

## Materials and methods

### FMR1 reference and patient DNA samples

29 lymphoblastoid cell line-derived genomic DNA samples (listed in Table 1) obtained from the Coriell Cell Repositories (CCR; Coriell Institute for Medical Research, Camden, NJ) were used for the initial optimisation and subsequent evaluation of direct triplet-primed PCR (dTP-PCR) assays. Additionally, a panel of archived and previously characterised peripheral blood-derived patient DNAs consisting of 54 NL, 25 PM and 28 FM *FMR1* carriers was tested in a blinded fashion. Approval to perform this study was granted by the National University of Singapore Institutional Review Board (07-123E) and the SingHealth Centralized Institutional Review Board (2013/-73/A).

**Table 1.** Genomic DNA samples from the Coriell Cell Repositories used for direct TP-PCR MCA and CE

Coriell ID	Sex	Coriell *FMR1* size	Results from a Consensus study (Ref. 47)	Direct TP-PCR		
				MCA result	Allele size	Allele structure
GM 07175	F	23/30	n.a.	NL	23/30	13 + 9/10 + 9 + 9
GM 06890	M	30	n.a.	NL	30	10 + 9 + 9
NA 07174	M	30	30	NL	30	10 + 9 + 9
GM 04479	F	n.a.	n.a.	NL	29/30	9 + 9 + 9/10 + 9 + 9
NA 07538	F	29/29	29/29	NL	29/29	9 + 9 + 9/9 + 9 + 9
GM 04738	M	n.a.	n.a.	NL	30	10 + 9 + 9
NA 20243	F	29/41	29/41	NL	29/41	9 + 9 + 9/10 + 9 + 20
NA 20238	F	29/30	29/30	NL	29/30	9 + 9 + 9/9 + 9 + 10
NA 20244	M	41	41	NL	41	9 + 9 + 21
NA 20234	F	31/46	31/46	IN	31/46	10 + 9 + 10/9 + 9 + 13 + 12
NA 20232	M	46	46	IN	46	9 + 36
NA 20235	F	29/45	29/45	IN	29/45	9 + 9 + 9/10 + 34
NA 20236	F	31/53	31/53	IN	31/54	10 + 9 + 10/pure
NA 20230	M	53	53	IN	54	Pure
CD 00014	M	56	56	IN	56	9 + 9 + 36
NA 20231	M	76	76	Ex	78	10 + 67
NA 20242	F	30/73	30/73	Ex	30/74	10 + 9 + 9/9 + 9 + 54
GM 06892	M	93	86	Ex	93	10 + 82
NA 20240	F	30/80	30/80	Ex	30/87	10 + 9 + 9/pure
GM 06907	F	29/85	n.a.	Ex	29/91	9 + 9 + 9/10 + 80
GM 06896	F	23/95-120-140	n.a.	Ex	23/115	13 + 9/10 + 104
GM 06891	M	118	No consensus	Ex	~160	Pure
GM 06852	M	>200	n.a.	Ex	>200	Pure
GM 07862	M	501–550	n.a.	Ex	>200	Pure
GM 07294	M	n.a.	n.a.	Ex	>200	Pure
GM 20239	F	20/183–193	20/no consensus	Ex	20/~200	10 + 9/pure
GM 07063	F	n.a.	n.a.	Ex	32/>200	9 + 22/pure
GM 05855	F	n.a.	n.a.	Ex	34/>200	10 + 23/pure
GM 07537	F	28–29/>200	n.a.	Ex	29/>200	9 + 9 + 9/pure

NL – normal; Ex – expanded; IN – indeterminate; pure – uninterrupted CGGs. n.a. – not available

### Direct TP-PCR and MCA

The dTP-PCR mix containing 1 X PCR buffer (Qiagen, Hilden, Germany), 2.5 X Q-solution (Qiagen), 0.1 X SYBR Green I nucleic acid dye (Roche Applied Science, Penzberg, Germany), 5 U HotStarTaq DNA polymerase (Qiagen), dNTP mix at a final concentration of 2 mM with a 5:1 dGTP and dCTP to dATP and dTTP ratio (Roche Applied Science), 100 ng of genomic DNA, and 0.60 μM each of primers, ‘F’ described by Fu YH et al. (Ref. 3), tail (5′TGCTCTGGACCCTGAAGTGTGCCGTTGATA3′), and a 1000-fold diluted TP-primer (5′TGCTCTGGACCCTGAAGTGTGCCGTTGATA[CGG]_5_3′) was prepared in a 15-μl reaction volume. An initial denaturation at 95°C for 15 min was followed by 40 cycles of 99°C for 45 s, 55°C for 45 s and 70°C for 8 min, and then a final extension at 72°C for 10 min on the GeneAmp^®^ PCR System 9700 (Applied Biosystems-Life Technologies, Carlsbad, CA). Subsequently, the amplicons were transferred into a 96-well plate for MCA on the LightCycler^®^ 480 Real-Time PCR System (Roche Applied Science) with a similar MCA program setting as described previously (Ref. 41). The dTP-PCR MCA results were analysed on the LightCycler^®^ 480 software and the melt peak temperature or *T*_m_ (the temperature at which the maximum −d*F*/d*T* value is recorded) obtained for each sample was rounded off to the nearest 0.05°C.

To identify *FMR1* expansion carriers among the male and female samples tested, two IM reference male (NA20232, 46 repeats and NA20230, 54 repeats) and two IM reference female (NA20234, 31/46 repeats and NA20236, 31/54 repeats) samples from CCR were included in each MCA plate/run as internal reference controls, respectively. While samples that generated MCA profiles with low *T*_m_ relative to the 46-repeat internal reference control were classified as ‘NL’, those that produced MCA profiles with higher *T*_m_ relative to the 54-repeat internal reference control were classified as ‘Expansion Carriers’, who require confirmatory assessment using reference method(s). In comparison, samples that displayed *T*_m_ identical to either one of the internal reference controls and those that generated MCA profiles in the ‘Indeterminate Zone’ (defined as the melting domain between the 46-repeat and the 54-repeat internal reference controls) are presumed to be ‘IM’, although additional testing is recommended to confirm their *FMR1* genotypes.

### Direct TP-PCR and CE

The dTP-PCR mix for sizing assay had a reaction setup identical to that described for MCA, except that SYBR Green I dye was excluded; 5′ end of the flanking F primer was labelled with 6-carboxyfluorescein (*Fam*); and 5 U of Taq Extender™ PCR Additive (Agilent Technologies, Santa Clara, CA) was included. An initial denaturation at 95°C for 15 min was followed by 40 cycles of 99°C for 45 s, 55°C for 45 s and 70°C for 8 min with a 15 s auto-extension at each cycle, and a final extension at 72°C for 10 min on the GeneAmp^®^ PCR System 9700 (Applied Biosystems). A 4-μl aliquot of *Fam*-labelled TP-PCR amplicon was mixed with 0.5 μl of MapMarker1000^®^ (BioVentures, Murfreesboro, TN) and 9 μl of Hi-Di™ Formamide (Applied Biosystems), denatured at 95°C for 5 min and subjected to CE (36 cm, POP-7™, 18 s 1.2 kV injection, 50 min 15 kV run) on the 3130*xl* Genetic Analyzer (Applied Biosystems). GeneScan electropherograms were analysed using GeneMapper^®^ software (Applied Biosystems, version 4.0).

The dTP-PCR electropherograms generally reflect both *FMR1* CGG repeat size and the distribution pattern of AGG interruptions. For instance, in case of NL samples with AGG-interrupted *FMR1* alleles, discrete *Fam*-labelled dTP-PCR peak clusters separated by clear zones of ~18 bp are expected owing to the poor annealing of TP-primers over AGG interruptions. In marked contrast, samples carrying PM and FM expansions display a continuous series of uninterrupted *Fam*-labelled dTP-PCR peaks differing by 3-bp from each other, a pattern characteristic of *FMR1* alleles with one or no AGG interruption. For FM samples, in addition to the uninterrupted *Fam*-labelled dTP-PCR peaks exceeding 700 bp, we also observed a FM-peak around 1050 bp. The dTP-PCR peaks in the range of 700–750 bp had signal intensities of 10–20 RFU, whereas the FM-peaks had signal intensities ranging from 30–80 RFU.

## Results

### Performance optimisation and evaluation of dTP-PCR assays on fragile X reference DNA samples

**Figure 1.**
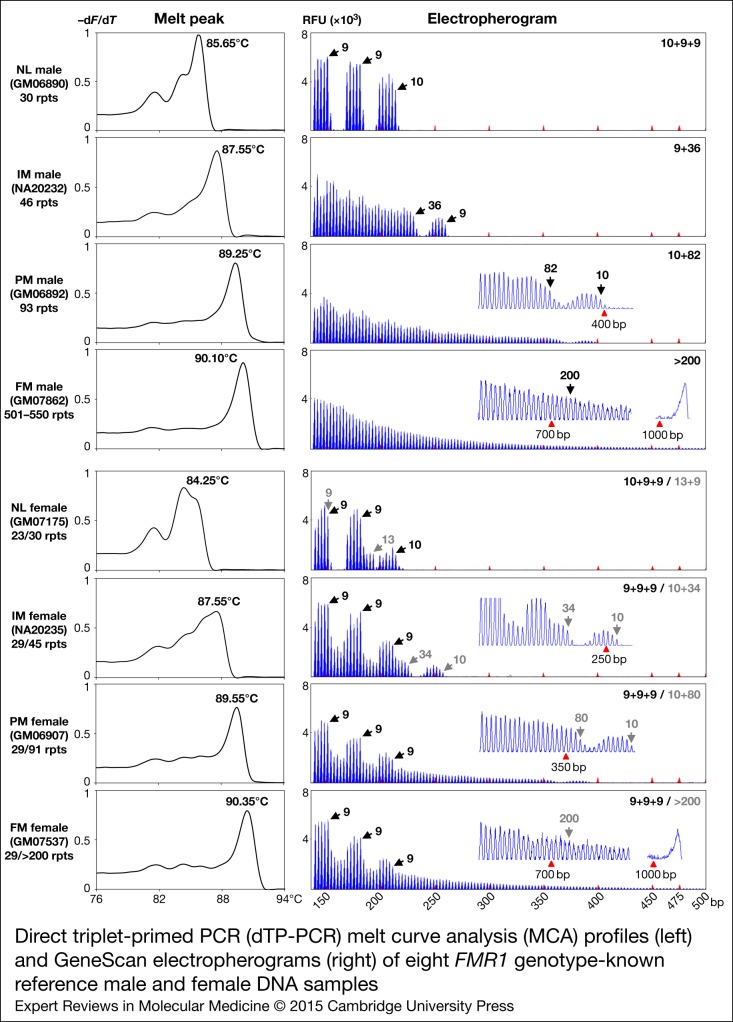
dTP-PCR MCA profiles (left) and GeneScan electropherograms (right) of eight *FMR1* genotype-known reference male and female DNA samples. Coriell IDs and CGG repeat sizes of the samples are indicated on the left and the melt peak temperatures (*T*_m_) are indicated on the MCA profile of each sample. The −d*F*/d*T* values are shown on the *y*-axis and the temperatures (°C) are shown on the *x*-axis. Distribution pattern of AGG interruptions within the CGG repeat region are shown on the top right corner of each dTP-PCR GeneScan electropherogram, where a ‘+’ sign represents an AGG interruption. Number of CGG repeats is indicated by numbered black and grey arrows. Red arrowheads in the inset panels indicate the base-pair (bp) size, and the red peaks in the main panel are from a ROX-labelled internal size calibrator, whose bp sizes are indicated at the bottom of the electropherogram panel. rpts: total number of CGG repeats including AGG interruptions.

We chose four male and four female Coriell Cell Repository (CCR) samples with NL, IM, PM and FM *FMR1* alleles for the initial optimisation of dTP-PCR assay conditions (Fig. 1). Carriers and noncarriers of *FMR1* expansion generated MCA profiles with distinctive melt peak temperatures (*T*_m_); both NL (GM06890 and GM07175) and IM (NA20232 and NA20235) samples produced MCA profiles with *T*_m_s that ranged from 84.25 to 87.55°C, while expanded PM (GM06892 and GM06907) and FM (GM07862 and GM06852) samples yielded MCA profiles with a pronounced shift in their *T*_m_ to 89.25°C or above (Fig. 1, left). Moreover, the dTP-PCR MCA profiles showed good correlation with the CE estimated CGG repeat size and structure, which is well-illustrated across both male and female samples with different *FMR1* genotypes (Fig. 1, right).

**Figure 2.**
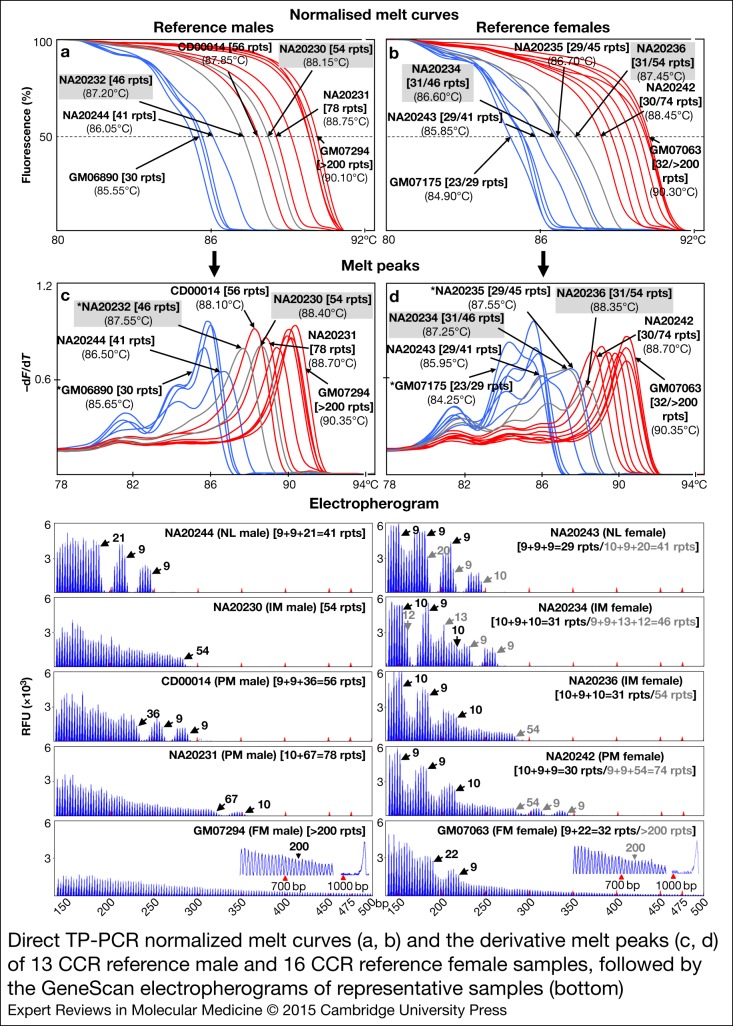
Direct TP-PCR normalised melt curves (a, b) and the derivative melt peaks (c, d) of 13 CCR reference male and 16 CCR reference female samples, followed by the GeneScan electropherograms of representative samples (bottom). Grey melt curves and peaks indicate the MCA profiles of the internal reference controls. GeneScan electropherograms of samples marked with asterisk (*) are shown in Figure 1.

Furthermore, to determine the ability of dTP-PCR assays in accurately categorising samples carrying *FMR1* alleles of different CGG repeat sizes and AGG interruption patterns, we carried out a preliminary performance assessment study on 21 additional CCR reference DNAs, and also re-evaluated the eight samples used for initial assay optimisation (listed in Table 1). Figure 2 presents the dTP-PCR MCA data of 13 reference males and 16 reference females as normalised melt curves (a, b) and melt peaks (c, d), followed by the GeneScan electropherograms of representative samples. Using MCA we ascertained the *FMR1* genotype class of each sample based on its *T*_m_ relative to reference controls that mark the lower and upper limits of the ‘Indeterminate Zone’. For the analysis of the reference males, the melt curve and peak temperatures of the IM male samples NA20232 and NA20230 marked the ‘Indeterminate Zone’ boundary, whereas the IM female samples NA20234 and NA20236 were selected to define the ‘Indeterminate Zone’ for the analysis of the reference females. As expected, all NL males carrying *FMR1* alleles ranging in size from 30 to 41 CGG repeats, displayed lower *T*_m_s compared with that of NA20232 carrying a 46-repeat *FMR1* allele with a 9 + 36 repeat-pattern, where ‘+’ indicates the position of AGG interruption relative to CGG repeats. In marked contrast, most PM and all FM males generated right-shifted MCA profiles that displayed higher *T*_m_s compared with that of NA20230 carrying a 54-repeat *FMR1* allele with no AGG interruptions. A PM male (CD00014) generated a TP-PCR melt peak *T*_m_ in the ‘Indeterminate Zone’. CD00014 carries a sequence-verified 56-repeat allele (the second smallest PM allele size) with a 9 + 9 + 36 interruption pattern, which was also confirmed by its CE pattern. With the exception of this PM male, all NL, PM and FM reference males were unambiguously classified by MCA as nonexpanded or expanded.

The *T*_m_s derived from the MCA profiles of reference female samples generally showed good correlation with the CGG repeat size of the largest *FMR1* allele present in the sample, except in case of GM07175, a NL female whose two alleles differ by seven CGGs. Consistently, this NL female displayed a melt peak *T*_m_ consistent with the shorter 23-repeat allele rather than the 30-repeat allele. However, an analysis of the peak pattern indicates the presence of a shoulder with a peak *T*_m_ consistent with the 30-repeat allele. Whether the *T*_m_ of the tallest melt peak or the higher *T*_m_ of the peak shoulder is chosen, this sample was classified appropriately. Importantly, the *FMR1* genotypes of all reference female samples were accurately classified by MCA. All NL females generated MCA profiles of lower *T*_m_s compared with that of NA20234 carrying a 46-repeat *FMR1* allele with a 9 + 9 + 13 + 12 repeat-pattern, whereas expanded PM or FM females produced MCA profiles of higher *T*_m_s compared with that of NA20236 carrying a 54-repeat *FMR1* allele with no AGG interruptions. A 45-repeat IM female (NA20235) displayed a slightly higher *T*_m_ relative to NA20234 and generated MCA profile in the ‘Indeterminate Zone’, presumably due to the presence of only 1 AGG interruption compared with the three AGGs in NA20234.

In addition, we have also demonstrated the utility of normalised melt curves with temperature differentials that are slightly different from the *T*_m_ of melt peaks. Similar to melt peaks, each normalised melt curve displayed a clear and distinct profile pattern of high resolution with respect to temperature and melt curve position relative to other CGG repeat alleles.

### Analysis of AGG interruption patterns and its effects on MCA profiles

To determine the reliability of dTP-PCR CE assay in accurately characterising AGG interruption patterns, we verified the repeat sequences of several reference males (GM06890, NA20244, NA20232, NA20230, CD00014, NA20231 and GM06892), a homozygous reference female (NA07538) and the IM alleles of two reference females – NA20235 and NA20234 by Sanger sequencing (data not shown). Generally, we observed complete concordance between most Sanger sequencing and dTP-PCR CE results. Although the assay displayed superior performance in male samples, characterising the distribution pattern of AGG interruptions of some heterozygous female samples was challenging; this is best-illustrated in case of NA20234 carrying a 31-repeat NL allele and a 46-repeat IM allele with 10 + 9 + 10 and 9 + 9 + 13 + 12 repeat-patterns, respectively (shown in the electropherograms in Fig. 2).

In addition, we also examined the MCA profiles of reference samples with identical CGG repeat size, but different AGG interruption patterns. In the IM samples, NA20230 and NA20236 carrying a pure 54-repeat *FMR1* allele, only a negligible *T*_m_ variation of 0.05°C was observed. In contrast, we observed a *T*_m_ difference of 0.30°C between NA20232 and NA20234, both of which carried an *FMR1* allele with 46 CGG repeats, with lower *T*_m_ recorded for NA20234 harbouring 3 AGG interruptions compared with NA20232 harbouring only one AGG interruption. This observation is consistent with our previous study, wherein we reported varied melt profiles among samples differing only in their repeat-patterns (Ref. 41).

### Detection of low-level mosaicism for expanded *FMR1* allele in artificial DNA mixtures

**Figure 3.**
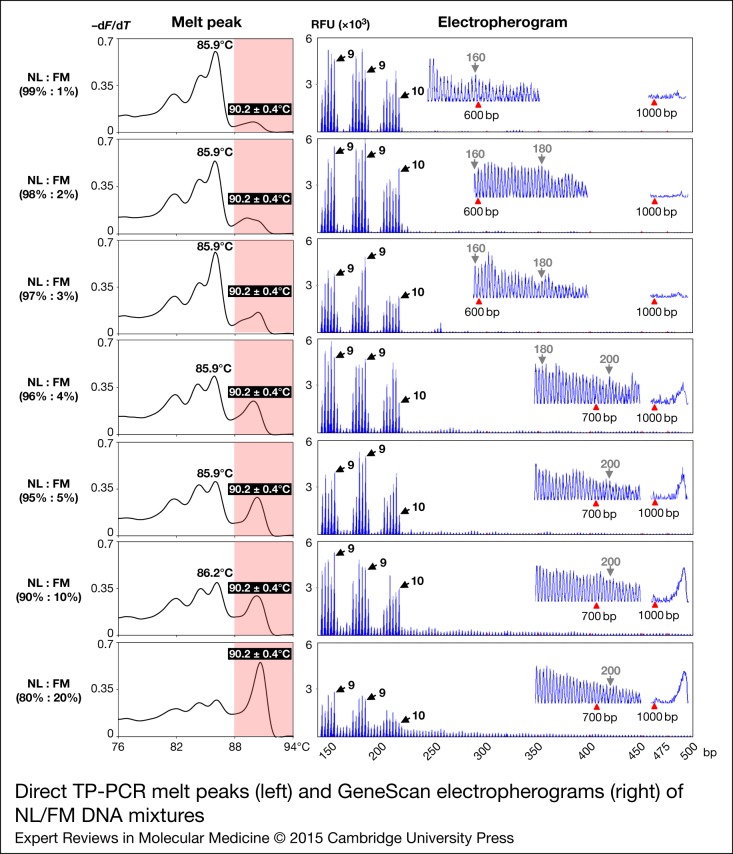
Direct TP-PCR melt peaks (left) and GeneScan electropherograms (right) of NL/FM DNA mixtures. Presence of FM allele in the NL/FM DNA mixtures was confirmed by the identification of MCA peaks with higher *T*_m_ in the melting domains highlighted in pink.

The sensitivity of dTP-PCR assays in detecting low-level mosaicism for an expanded FM allele was tested using artificial DNA mixtures that contained 1, 2, 3, 4, 5, 10 and 20% of the FM allele. To generate these NL/FM DNA mixtures, we pooled the genomic DNAs of GM06890 (NL male, 30 repeats) and GM07862 (FM male, ~501–550 repeats) in different proportions with the total DNA input maintained constant at 100 ng. The dTP-PCR MCA of NL/FM DNA mixtures revealed efficient amplification of FM allele in the presence of predominant levels of NL allele, and enabled accurate detection of FM mosaicism from as low as 1% (Fig. 3, left). The low-level mosaicism for FM allele allowed visualisation of both NL and FM MCA peaks with discrete *T*_m_ in 1–10% NL/FM DNA mixtures. The availability of more FM template in the 20% NL/FM DNA mixture resulted in a much more prominent FM MCA peak that closely resembles the melt profile of a typical FM female. We noticed a slight reduction in the sensitivity of the dTP-PCR CE assay compared with MCA, with FM mosaicism detected down to 4% (Fig. 3, right).

### Blinded validation of dTP-PCR assays on clinical samples

**Figure 4.**
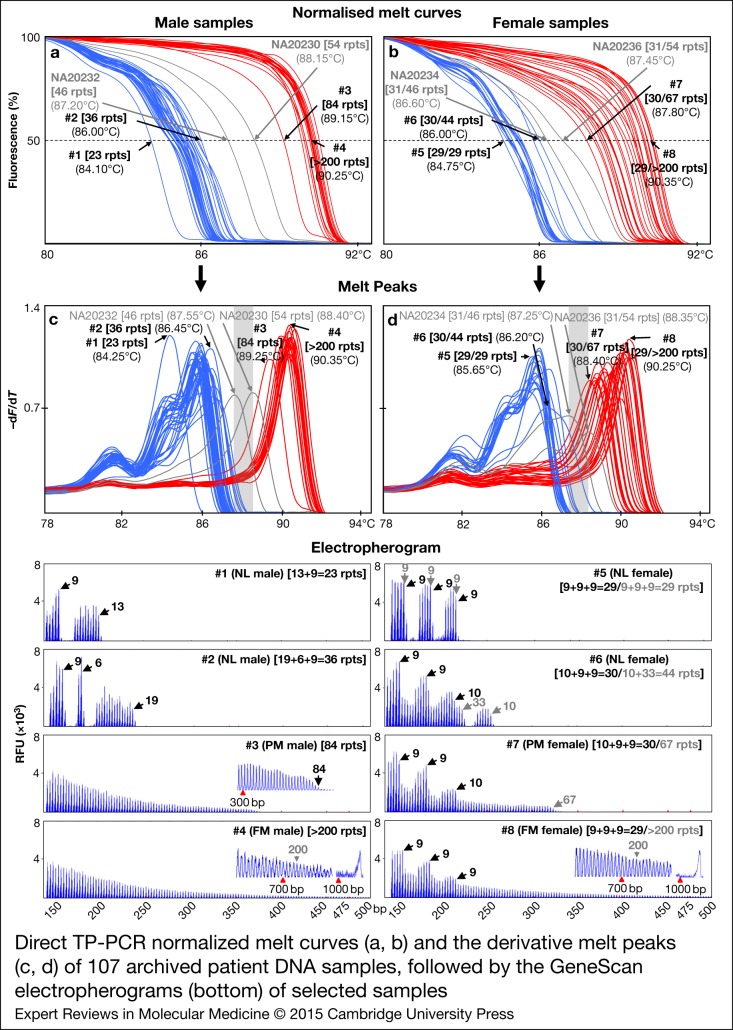
Direct TP-PCR normalised melt curves (a, b) and the derivative melt peaks (c, d) of 107 archived patient DNA samples, followed by the GeneScan electropherograms (bottom) of selected samples. MCA profiles of samples carrying NL and expanded *FMR1* alleles are clustered to the left and right of the ‘Indeterminate Zones’ (highlighted in grey), respectively.

A total of 107 clinical samples, previously characterised by PCR and/or Southern analysis, were included in the blinded validation study of dTP-PCR assays. The patient cohort comprised 54 NL and 56 PM and/or FM expansion carriers, and had no samples with *FMR1* alleles in the IM size range. In addition, we also included two sets of internal reference controls from CCR, NA20232 and NA20230, and NA20234 and NA20236 to define the cut-off temperature ranges for male and female samples, respectively. Normalised melt curves and melt peaks of all clinical samples with internal reference controls, followed by the GeneScan electropherograms of representative NL, PM and FM carriers are shown in Figure 4. As expected, in both males and females, two clusters of MCA profiles, one from NL samples exhibiting lower *T*_m_ compared with the respective 46-repeat reference control, and another from samples carrying expanded *FMR1* alleles with *T*_m_ higher than that of the respective 54-repeat reference control, were generated. Consistent with the lack of IM samples, no MCA profiles were detected in the ‘Indeterminate Zone’. In general, we observed complete agreement between MCA and CE results for all clinical samples and achieved 100% sensitivity, 100% specificity, 100% positive predictive value and 100% negative predictive value for both dTP-PCR assays. Additionally, we verified the CGG repeat size and AGG interruption pattern of the *FMR1* alleles of 17 NL males by Sanger sequencing and detected absolute concordance with dTP-PCR CE results (data not shown).

## Discussion

One common current approach to FXS molecular diagnosis involves a combinatorial strategy of employing simple PCR and CE to identify putative expansion carriers in the first instance, followed by the SB analysis or the most recent mPCR-CE (Ref. 40) for additional characterisation of samples carrying expanded *FMR1* alleles. These first-tier PCR-based fragile X testing methods perform remarkably well in identifying *FMR1* expansions and significantly reducing the need for labour-intensive approaches such as the gold standard SB analysis (Refs 37, 38). However, for large-scale universal newborn and carrier screening applications, methods relying on CE might not be ideal first-line screening tools.

Alternatively, Tassone et al. (Ref. 35) proposed a two-stage sequential approach of conventional repeat-spanning PCR followed by a chimeric PCR that utilises a CGG-repeat annealing primer, whose utility in newborn screening was recently demonstrated (Ref. 8). The first round PCR screen identified males with *FMR1* alleles of up to ~330 CGG repeats and apparent heterozygous females of up to a PM size range of at least ~160 CGG repeats (Ref. 42), but a second round PCR screen was necessary to distinguish NL homozygous females from heterozygous females carrying a nonamplifiable FM allele. This two-step strategy ultimately minimised the number of samples that required SB analysis.

In this study, we have presented an alternative FXS screening strategy that relies on the analysis of amplicon melt characteristics. The proof-of-principle of this strategy was first demonstrated in 2012 (Ref. 41). This strategy utilises direct triplet-primed PCR melting curve analysis (dTP-PCR MCA), which is both cost-effective and reliable in identifying *FMR1* expansions. A major improvement over the method of Teo et al. (2012) is that our improved assay identifies PM and FM carriers based on melt peak temperature (*T*_m_) instead of the resumed baseline temperature, thus significantly improving accuracy of temperature determination. In addition, we also evaluated the concordance between MCA results and the actual genotypes of samples through a direct comparison of *T*_m_ with *FMR1* CGG repeat size.

Initially, we assessed the performance of the improved MCA-based screening tool on 29 CCR (Coriell Cell Repositories) reference DNA materials using four internal reference controls of known CGG-repeat size. Male and female reference samples were analysed relative to two different sets of controls, each with a 46-repeat and a 54-repeat reference sample, which roughly defined the melting domain of IM *FMR1* alleles or the ‘Indeterminate Zone’. In these reference samples with *FMR1* alleles spanning the entire spectrum of fragile X mutation, the first-line MCA screen identified PM and FM expansion carriers with high sensitivity and specificity. Among the reference males, one PM carrier (CD00014) harbouring a 56-repeat *FMR1* allele with a 9 + 9 + 36 repeat-pattern was identified in the ‘Indeterminate Zone’ and was therefore marked for further analysis. This allele harbours two AGG interruptions which may have lowered its *T*_m_ relative to the 54-repeat control sample, which lacks AGG interruptions. This emphasises the need to have a cut-off temperature range rather than relying on the cut-off temperature of a single internal reference control. For instance, if the analysis was only based on the cut-off temperature of the 54-repeat control, this small PM male would have been wrongly classified as NL or IM, resulting in a false-negative call and reduced assay sensitivity. Conversely, in the presence of the 46-repeat control alone, most IM samples may appear as expanded, which would result in high false-positive calls and reduced assay specificity.

Remarkably, there was complete agreement between the MCA melt peak temperatures and CE sizing results of the dTP-PCR amplicons with respect to the classification of samples as ‘nonexpanded’ or ‘expanded’. The direct TP-PCR CE assay enabled sizing of *FMR1* alleles and facilitated determination of AGG interruption patterns, both of which are crucial for predicting the risk of unstable *FMR1* CGG-repeat expansions during maternal transmission (Ref. 25). Since the first report by Eichler et al. (Ref. 24), several studies have highlighted the utility of maternal CGG-repeat size (Refs 23, 43, 44) and the density of AGG triplets (Refs 27, 45, 46) in ascertaining the risk of *FMR1* allelic expansions. These findings argue for mapping AGG interruptions within the CGG-repeat tract, information that can be potentially used to predict the likelihood of expansion events among women carrying at-risk *FMR1* alleles in the IM and PM size ranges. The dTP-PCR CE assay has potential applications in studies aiming to characterise AGG interruption patterns, although interpretation of the repeat structures can be slightly difficult in heterozygous females with complex repeat structures due to the overlap of amplicon peaks from both the *FMR1* alleles.

We generally observed good agreement between the *FMR1* allele sizes determined by dTP-PCR CE and the sizes reported by CCR, except for discrepancies in the PM samples GM06891 and GM20239. We detected a PM allele of ~160 CGG repeats in the cell-line derived genomic DNA of GM06891, contrary to the previously reported size of 118 CGG repeats in this PM male (Ref. 47). However, our observation is in agreement with a recent study that reported instability and expansion of the PM allele in the same lymphoblastoid cell line (Ref. 48). In GM20239, we observed a slightly larger expanded allele of ~200 CGG repeats instead of the reported PM size range of 183–193 CGG repeats. Again, our observation is consistent with another report that sized the largest expanded allele as ~206 CGG repeats (Ref. 38).

In the artificial NL/FM DNA mixtures, the MCA assay detected FM mosaicism down to 1%. This high degree of sensitivity is critical for a first-line screening tool, without which samples with low-level mosaicism for PM or FM might not be detected. In the blinded patient DNAs, including NL and expanded PM and FM samples, the MCA screen accurately classified all samples with 100% sensitivity and specificity. Such an exceptional performance of the screening assay may be partly attributed to a lack of IM samples in the patient cohort, which was not intentional but reflects a very low frequency of IM alleles in Asian populations (Refs 49, 50, 51, 52, 53). It is thus important to note that in populations with higher frequency of IM alleles, the specificity (and even possibly sensitivity) of this screening tool might be lower than 100%.

The MCA method has potential applications as a first-line tool in newborn and carrier screening programs owing to its simple design and flexibility to tailor the approach for different purposes. For NBS of FXS, utilising IM internal reference controls would mean identifying both PM and FM carriers. Identification of PM babies might raise ethical concerns related to detection of risk for late-onset disorders, although recent reports of PM carriers with developmental delay suggest a possible benefit to PM screening in babies, that of early developmental intervention (Ref. 21). If the aim is to identify both PM and FM, the demonstrated approach with IM internal reference controls will ensure high sensitivity and specificity in identifying expansion carriers.

Alternatively, for identifying only FM, an appropriate PM reference control can be used to minimise the number of false positives. Nevertheless, all the ‘false positives’ identified will be eventually excluded at the allele sizing second stage. If the population PM allele frequency is high, a higher false-positive FM rate can be expected following the first-tier MCA screen. Nonetheless, this method would still compare well with a first-tier screen employing flanking PCR and agarose gel electrophoresis (Ref. 35), whereby ~40% of females will require second-tier testing due to apparent homozygosity for the NL allele. Using PM reference controls, it should also be possible to avoid identification of IM carriers, whose involvement with disease are still in contention.

For population carrier screening programs aimed at identifying PM women of reproductive age, the use of IM internal reference controls would enable identification of virtually all but the smallest PM allele carriers, who are most likely to display melt profiles in the ‘Indeterminate Zone’. This method also has potential applications as a first-line screen for FXS in a diagnostic setting. Conceivably, in a population with low prevalence of FM alleles, a majority of tested samples will be negative for FM expansions even in high-risk groups such as children with intellectual/behavioural disabilities, and this screen could be a cost-effective first-tier tool to exclude ‘nonexpanded’ individuals. However, in an extreme hypothetical population wherein 1 of every two to three samples tested is positive for a FM allele, a screening assay such as this would obviously be unwarranted.

We calculated the reagent cost for fluorescent dTP-PCR, and the reagent plus consumables cost for amplicon CE on the ABI 3130*xl*, to be ~US$ 4.65 and ~US$ 2.30 per sample, respectively. In contrast, it costs ~US$ 3.05 and ~US$ 0.15 per sample to perform a nonfluorescent dTP-PCR and subsequent MCA assay on the Roche LC480, respectively. Although CE-based amplicon analysis allows for precise repeat sizing, employing it as a first-tier screening tool may not be cost-effective, especially in a large-scale population-based screening setting wherein a vast majority of the screened samples will be negative for *FMR1* expansions. Also, while some recent studies have demonstrated the technical feasibility of using CE to analyse hundreds of samples (Refs 9, 10), it is important to consider that such an approach might become very expensive when tens to hundreds of thousands of samples are tested annually in each jurisdiction.

Based on the known prevalence rates of *FMR1* alleles in the general population (Refs 9, 10), it can be estimated that for every 50 000 samples screened, ~48 500 samples (97%) will yield a negative result when tested for the presence of *FMR1* alleles of >45 CGG-repeats. It would cost US$ 160 000 to screen 50 000 samples using dTP-PCR MCA as a first-tier screening tool to exclude ‘nonexpanded’ samples, and another US$ 10 425 for dTP-PCR and CE confirmation analysis on an estimated 1500 screen-positive samples. If all 50 000 samples were to be screened directly by fluorescent dTP-PCR and CE analysis, it would cost US$ 177 075 more, or ~US$ 3.55 more per sample screened. Therefore, in a large-scale screening setting, exclusion of the majority of samples as ‘nonexpansion carriers’ using the dTP-PCR MCA assay, with more precise sizing confirmation of only the screen-positive samples by fluorescent dTP-PCR and CE analysis, could be a more cost-effective alternative to direct fluorescent dTP-PCR and CE analysis of all samples. Even in an unlikely hypothetical situation in which half the population carries an *FMR1* expansion, performing dTP-PCR MCA screening followed by the repeat-size confirmation of screen-positive samples using fluorescent dTP-PCR CE would still cost less than directly performing fluorescent dTP-PCR CE on all samples.

Unlike the approach of performing the dTP-PCR and MCA on the same real-time PCR instrument described in our previous study (Ref. 41), the current dTP-PCR assay was optimised on a regular thermal cycler followed by melt analysis on LightCycler^®^ 480 Real-Time PCR System (LC480). The rationale for proposing this strategy was to enable a higher throughput and reducing the turnaround time per sample. For instance, it is possible to process 16 plates of 96 samples by performing dTP-PCR on multiple inexpensive thermal cyclers in parallel and performing the amplicon melting for the 16 plates on a single LC480 instrument in ~24 h. In contrast, for 16 plates to be amplified and analysed completely on the LC480 would require ~6 days for one instrument or six LC480 s for the results to be out in 24 h. However, this method can also be carried out completely on the LC480 if desired (data not shown).

In summary, the dTP-PCR MCA method has potential applications as a first-line test to identify *FMR1* expansions in both males and females. In large-scale universal screening programs, ideally this screening test should be accompanied by a reference method, either SB analysis or mPCR-CE, for detailed characterisation of CGG-repeat size and methylation state of samples that test positive for an expansion in the first round MCA screen.
